# A Non-Destructive System Based on Electrical Tomography and Machine Learning to Analyze the Moisture of Buildings

**DOI:** 10.3390/s18072285

**Published:** 2018-07-14

**Authors:** Tomasz Rymarczyk, Grzegorz Kłosowski, Edward Kozłowski

**Affiliations:** 1 University of Economics and Innovation in Lublin, Research & Development Centre Netrix S.A., 20-209 Lublin, Poland; tomasz@rymarczyk.com; 2 Faculty of Management, Lublin University of Technology, 20-618 Lublin, Poland; e.kozlovski@pollub.pl

**Keywords:** inverse problem, electrical tomography, moisture inspection, dampness analysis, machine learning, nondestructive evaluation

## Abstract

This article presents the results of research on a new method of spatial analysis of walls and buildings moisture. Due to the fact that destructive methods are not suitable for historical buildings of great architectural significance, a non-destructive method based on electrical tomography has been adopted. A hybrid tomograph with special sensors was developed for the measurements. This device enables the acquisition of data, which are then reconstructed by appropriately developed methods enabling spatial analysis of wet buildings. Special electrodes that ensure good contact with the surface of porous building materials such as bricks and cement were introduced. During the research, a group of algorithms enabling supervised machine learning was analyzed. They have been used in the process of converting input electrical values into conductance depicted by the output image pixels. The conductance values of individual pixels of the output vector made it possible to obtain images of the interior of building walls as both flat intersections (2D) and spatial (3D) images. The presented group of algorithms has a high application value. The main advantages of the new methods are: high accuracy of imaging, low costs, high processing speed, ease of application to walls of various thickness and irregular surface. By comparing the results of tomographic reconstructions, the most efficient algorithms were identified.

## 1. Introduction

This article presents the results of research on the development of an effective and non-invasive method for the detection of moisture walls and historical buildings. Humidity is one of the basic, and at the same time undesirable, physical characteristics of building materials [1]. Detection of water inside the walls of buildings and structures made of bricks or lightweight concrete and brick blocks is one of the most frequently performed tests.

All buildings are exposed to various factors that erode the material of which they are made. Such factors include water [2]. Many buildings have damp walls and foundations [3]. This is evidenced by often appearing molds and fungi, dark spots, and detachment of plasters or paint coatings. This is especially factual for older buildings. One of many reasons for this is the technology and materials that were formerly used in construction—for example, lack of insulation. Moisture in the walls significantly reduces their durability. The bricks and mortar that contain water are significantly weakened and are less resistant to compression, which is particularly true for lime mortar. This results in both deteriorations of the building’s operational conditions and safety. In addition, the water accumulated in the walls significantly worsens the thermal insulation properties of the walls and contributes to their gradual erosion. Another important problem related to moisture in walls is their harmful impact on inhabitant’s health. Microclimate, which arises in rooms and walls with high humidity, often causes the formation of mold fungi that can cause respiratory diseases and intoxication.

It can be argued that the main factor causing the destruction of the walls is just moisture. In combination with daily temperature changes, moisture has the greatest impact on the overall strength and durability of building structures. The water in the outer wall has a negative effect on the walls regardless of the state of aggregation, i.e., in solid form (ice, snow), as a liquid (rain) and gas (water vapor). Most structural defects, e.g., brick movements, cracking, molds, fungi, and chemical reaction, are initiated and compounded by the presence of moisture.

In order to reduce the risks associated with excessive humidity, moisture evaluations are necessary for buildings [4]. Such tests can be helpful in determining the impact of rainwater and groundwater, leakage, and moisture from water supply and sewage systems as well as condensation of water vapor.

**Figure 1:**
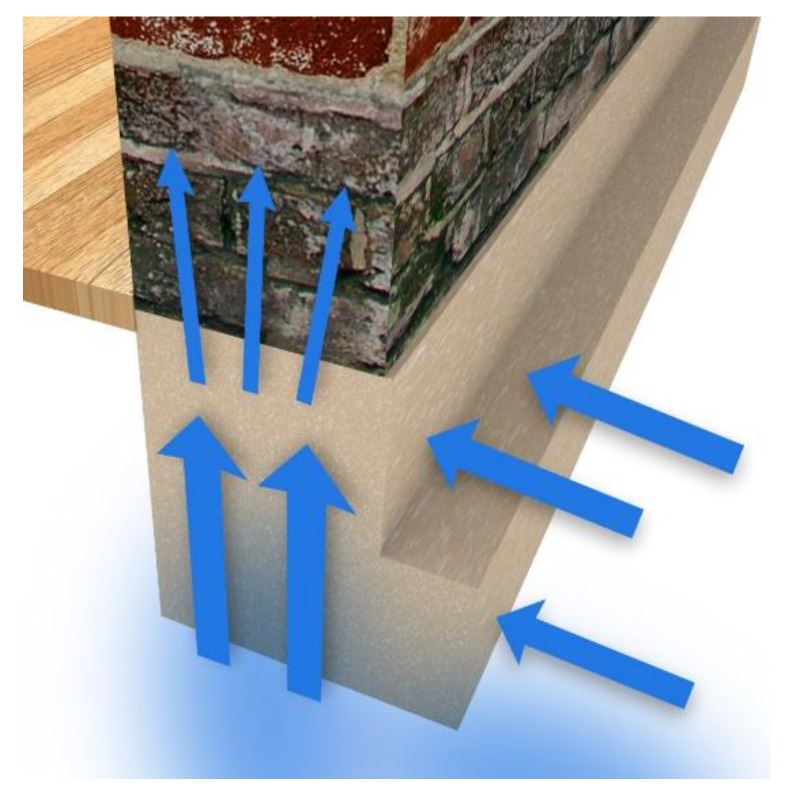
The rising moisture resulting from the direct connection of soil with masonry [14].

Permeation of moisture in the walls of old buildings that are in direct contact with the soil due to the lack of a horizontal or vertical barrier separating the walls from water in the soil leads to migration of moisture (Figure 1). This leads to the migration of dissolved in water salt, which is responsible for many construction problems. Building materials, both natural and man-made (e.g., the brick or concrete), are porous. Moisture from bricks and masonries can be drawn by gravity using the capillary effect [5].

The condition of effective prevention of moisture in the building walls is its proper identification. There are many methods that can generally be divided into two groups—destructive and non-destructive methods. For obvious reasons, non-destructive methods are more desirable and have bigger applied value [6,7]. This feature is gaining importance when walls’ humidity needs to be measured in buildings of historical importance. Among the destructive methods can be distinguished, among others, the “drying-weight” or “carbide” method. Unfortunately, this is an invasive examination. The destructive methods consist in taking samples of the material being examined, which is not always possible, especially in the case of historic buildings. In such cases, these types of methods are not recommended, because they involve a violation of the structure of the examined object. Therefore, non-invasive tests may be a better solution in such situations.

Non-destructive methods include, for example, thermovision or ultrasound methods. The disadvantage of thermovision is its exterior nature and the impossibility of penetrating under the surface of the investigated structure. The ultrasound approach is of little use due to the high porosity of materials containing cells (pores) filled with air or water.

Non-destructive methods also include electrical impedance tomography (EIT), in which electrical measurements are made [8]. This method, thanks to the measuring device and the implemented algorithms, allows for non-invasive spatial determination of the degree of moisture. In the case of impedance tomography, it is a technique for imaging the spatial distribution of conductivity [9].

**Figure 2:**
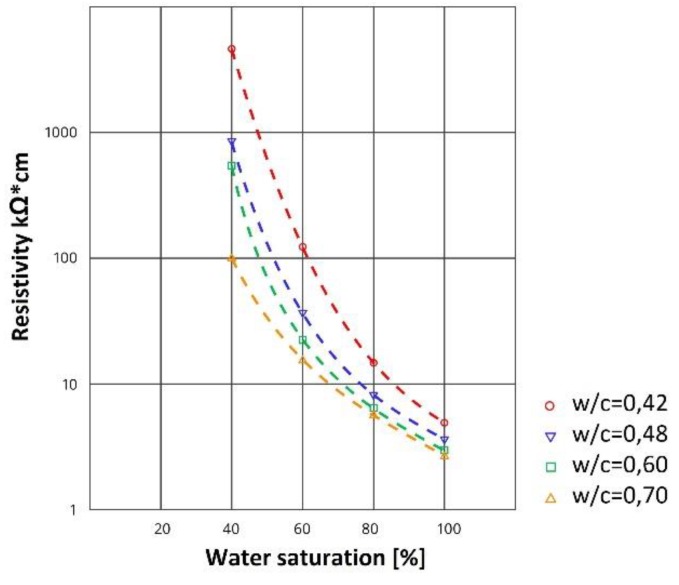
Relationship between saturation and resistivity of concrete [15].

Previous studies show that electrical resistance can be used to measure the humidity of concrete and masonry walls [10,11,12]. There is a known relationship between moisture inside a porous building material and its electrical resistivity (Figure 2). Similar relationships can be observed for a brick wall and a lightweight concrete blocks wall. The electrical resistance increases as the moisture content decreases. It can be seen that the smallest change in resistance occurs in the highest range of moisture content. Although current techniques for measuring moisture in concrete and brick walls are accurate, the use of electrical resistance has the advantage of being a relatively simple procedure that can be used with inexpensive equipment [13].

Each measuring technique has its own conditions, advantages, and disadvantages. Thanks to this it can be used only in special circumstances. Currently, the main problem in research on the concentration of moisture in the walls is the lack of a method that provides spatial imaging of its distribution inside the wall without the need to take samples. Most of the available research methods allow only a spot evaluation of moisture, which makes it possible to obtain only a discrete model [4]. In most cases, methods based on real data are invasive.

This fact is a basic problem with regard to the analysis of thick walls because the moisture inside each wall is usually a few percent higher than at its surface [16]. The destructive nature of currently used techniques requiring sample collection is unacceptable, especially in historical buildings. In such cases, only non-invasive methods may be used.

**Figure 3:**
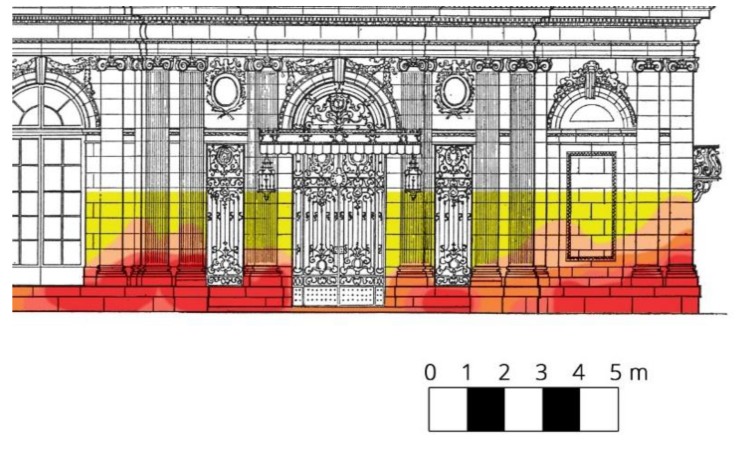
An image of building walls’ dampness developed on the basis of the results of an electrical test. Higher moisture concentrations are depicted by a color of higher intensity [17].

The humidity of the walls can be directly measured with electric meters. Electrical moisture meters, and in particular conductivity meters, are sensitive to very low amounts of moisture and/or some types of contaminants with soluble salt. For example, a free moisture content lower than 0.1% can cause high meter readings. Due to the influence of salinity causing a change in electrical resistance and a small depth of measurement, evaluations made with the use of electric humidity meters should be considered as not very accurate [5,16]. They can give an approximate image of the dampness inside the walls (Figure 3).

The main purpose of this study is to present and compare non-destructive algorithms based on electrical tomography that allow estimation of humidity not only on the wall surface but also inside the masonry wall.

With reference to the tomography of building walls, the most commonly used methods are impedance tomography (EIT), capacitance tomography (ECT), and resistive tomography (ERT). All the above methods belong to the group of electrical tomography methods, including many tomographic techniques showing the distribution of electrical parameters in the tested object [18,19,20], while the EIT shows the spatial distribution of conductivity γ [21,22]. Authors such as Holder or Karhunen et al. [23,24] in the monograph give the general principles of the EIT, its instruments, procedures, and challenges.

The proposed tomographic method in which the building materials humidity evaluation is an indirect assessment based on a different physical characteristic, such as resistivity, allows for many measurements (tomographic approach in [25,26]) without the need to damage the tested object.

The tomographic approach allows archiving the moisture distribution inside the wall in a digital form and comparing it with the next results in the future (moisture monitoring) when it is necessary. This action is extremely useful in buildings requiring the use of a high imaging efficiency method, in particular: constant monitoring of wall humidity, control of the effectiveness of the methods used for drying walls and assessment of the moisture condition of load-bearing walls, in particular, thick ones.

The main advantages of the proposed measurement system are the non-invasive and non-destructive measurement of the tested object thanks to specially designed electrodes, the possibility of imaging the moisture distribution not only on the surface but also inside the investigated object. The described research uses simulation tools based on the Matlab scientific software and scripts in the Python and R programming languages. A special role was played by the toolbox called EIDORS dedicated to the Matlab software [18]. It has been used for modeling domain and topological algorithms using the finite element method (FEM) to solve the inverse problem (IP).

The structure of the article was divided into five parts including the introduction. Section 2 presents the hardware of the measurement system and algorithms for solving forward and inverse problems. Section 3 presents the results of tests both in relation to simulation experiments and to the reconstruction of the real object. The analysis covered three types of algorithms applicable in EIT: LARS, ElasticNET and ANN. The real object was also reconstructed. Section 4 includes the comparison of three selected algorithms and discussions in the perspective of previous studies. It also refers to other, known methods within the studied issues. The possible improvements were also suggested. Finally, Section 5 summarizes this article.

## 2. Materials and Methods

This chapter presents a measurement system that enabled the collection of electrical data used subsequently to solve forward and inverse problems. Then there are short descriptions of four selected algorithms: Least Angle Regression (LARS), ElasticNet, Artificial Neural Networks (ANN), and Gauss-Newton. The first three methods were used in the experiments on tomographic imaging and compared with each other. All tested algorithms are classified as machine learning and artificial intelligence methods. Thanks to the above algorithms, it was possible to use the supervised machine learning method, which in combination with the parallel computing (multi-core CPU, GPU) allowed us to quickly reach effective solutions in the field of building models of reconstructed objects.

### 2.1. Selected Hardware Issues, Sample Models, and Reconstructions

Electrical tomography is a technique for imaging the distribution of conductivity or permeability inside an object under investigation based on measurements of the potential distribution on the object’s surface. Numerous different techniques can be used in the process of optimization of tomographic methods.

**Figure 4:**
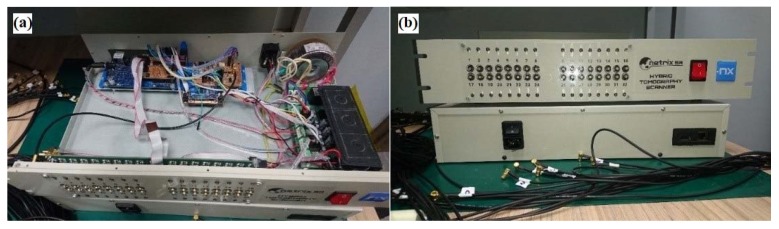
The measurement device: (**a**) inside view, (**b**) main panel view.

The data collection system collects the measured voltage from the electrodes and then processes the data. Conventional data acquisition systems require voltage, filtering, demodulation and converting equipment to be digitized and a signal processor to transfer data to a computer. Our device for measurement in electrical tomography uses two methods: electrical capacitance tomography and electrical impedance tomography. It allows you to take measurements up to 32 channels. Figure 4a shows the inside of the tomograph, while Figure 4b shows the main panel of the device with sockets designed to connect the electrodes. This device provides a non-invasive way to test the spatial distribution of moisture. The system includes an additional software solution. The advantage of the system is the simultaneous measurement of voltage and capacitance.

**Figure 5:**
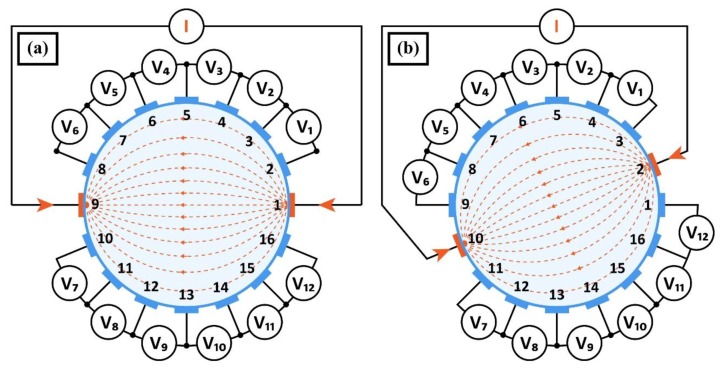
Voltage measurement method: (**a**) first measuring cycle, (**b**) second measuring cycle.

The measurements were made using the Polar GND EIT method, at 1 kHz frequency and 100 uA excitation current. An object whose internal electrical properties are unknown is surrounded by electrodes placed on its edge and electrically excited in various combinations (see Figure 5). Measurements are made for all possible ways of connecting the power source to the next pairs of electrodes. Other electrodes measure voltage drops. Both parts of Figure 5a,b shows two exemplary measuring cycles. In this way a series of measurements is created. For a system with 16 electrodes (we mark them as variable *n*), there are 16 possibilities to connect the power source, but because of the symmetry of the system, we accept only half of them (*n*/2 = 8).

Input data for the image construction algorithm are voltage measurements made between adjacent electrodes. Measurements made on electrodes with an attached excitation source are omitted due to unknown voltage drop between these electrodes and the tested area. For a system of *n* = 16 electrodes and any projection angle, *n* − 4 = 12 independent measurements can be obtained. Thus, the full number of voltages measurable between neighboring voltage electrodes at *n*/2 = 8 angles is: (*n* − 4) (*n*/2) = 12 × 8 = 96. The method of measuring the inter-electrode voltages shown in Figure 5 corresponds to the first and the second projection angle. For subsequent angles, sequential switching of the power supply and measurement circuit to the neighboring electrodes takes place.

For a system of *n* = 32 electrodes and any angle of projection, *n* − 4 = 28 independent measurements can be obtained. Hence the full number of possible independent measurements of voltages between neighboring voltage electrodes at *n*/2 = 16 angles is: (*n* − 4) (*n*/2) = 28 × 16 = 448.

Potential values of electrodes depend on the current distribution within the region, and thus also on the distribution of conductivity. The algorithm of computer image reconstruction is looking iteratively for such a distribution of conductivity, for which the calculated values of inter-electrode voltages are as close as possible to the corresponding measurement values.

The measured voltage is generally a voltage drop on the two impedances of the electrodes and the impedance of the object. The voltage drop on the impedances of the spot electrodes can be omitted due to the high impedance of the measuring system. In the case of surface electrodes, the potential decrease at the electrode is very low, the tested object has low conductivity, while the electrode has a high conductivity. Therefore, the voltage drop is negligible, the surface impedance coefficient of the electrode tends to zero (it is negligibly small), but it is programmatically included in the reconstruction process. The contact impedance is included in the model but has a limited effect on the measurement.

Voltage drops are measured on the surface of the tested object, so it is a non-invasive method. After applying the power source in the wall, current starts flowing, which has a higher value closer to the shore and the power source. The further away from the electrodes, the more the current flow is getting smaller (tends to zero). This is the factor that causes the reconstruction to be more optimal closer to the measuring electrodes, the further away from the electrodes, the detection precision may be lower (the reconstruction may be worse due to the current depth distribution). Therefore, measurements on one edge usually give worse quality compared to measurements on two or more edges. Sometimes, however, only one-sided measurement is possible.

**Figure 6:**
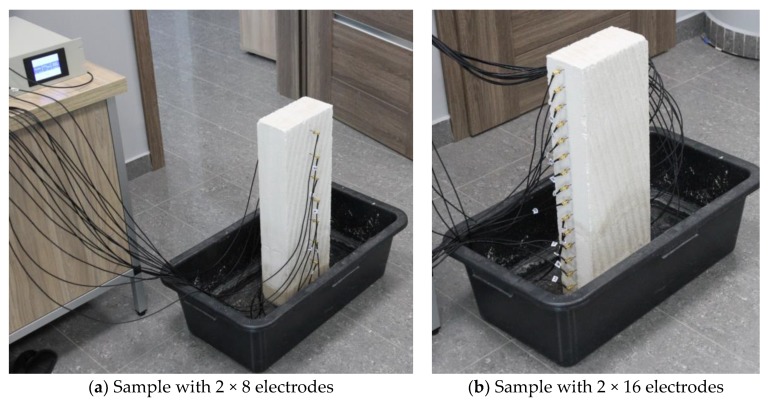
A tomographic laboratory to study the moisture inside the cellular lightweight concrete block.

The tested physical models of the wall parts contained 16 or 32 electrodes each for measuring the wet wall. The electrodes were placed on both sides of the tested wall sample. Electrical impedance tomography is based on the measurement of the potential difference. The ability to determine the wall condition results from the unique conductivity value of each material. The necessary equipment such as electrodes, meters, alternating current generator, multiplexer and a computer with LabVIEW and EIT modules was used for the measurements. Figure 6a,b shows the partially immersed lightweight concrete block on which the surface electrodes are placed. It can be seen that in the presented picture the block samples have 2 × 8 (a) and 2 × 16 (b) electrodes. 

As mentioned before, a big problem related to the moisture content testing is the lack of a method allowing us to determine its spatial distribution without the need to take samples. The method that enables this is electrical impedance tomography. It consists in evenly distributing the electrodes on the tested object and ensuring a good contact of their surface with the tested surface. Unfortunately, often the wall surfaces have a varied shape. Also, the building materials themselves, such as brick or plaster, have a certain porosity, which makes measurement difficult. To ensure an adequate flow of electric current between the individual electrode pairs, a special multilayer electrode was developed.

**Figure 7:**
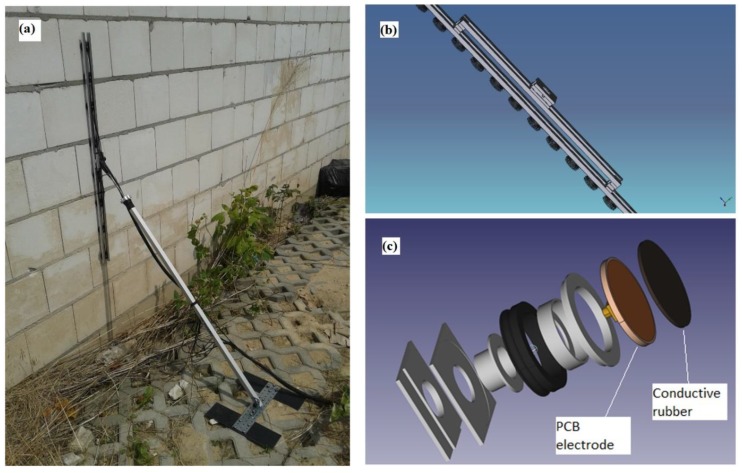
The concept of the electrodes: (**a**) way of fixing rubber electrodes to the building wall, (**b**) set of rubber electrodes, (**c**) structure of a single rubber electrode.

Ensuring the proper contact of the electrodes with the wall is particularly important in testing objects with an uneven as well as a rough surface. An example of the use of this type of electrodes is the moisture condition investigation inside the masonry. The development of effective and efficient measuring electrodes for impedance tomography has proved to be a serious challenge. In order to ensure optimal contact between the electrode and the wall, an electrode with a flexible contact surface and articulated mounting was designed. Figure 7a shows how to attach a set of rubber electrodes to the building wall. Figure 7b shows a view of the electrode set, while Figure 7c shows a schematic view of the structure of a single rubber electrode.

A complete electrode consists of three modules: a specific electrode, a PCB with a contact socket, and a fastening system. Mechanically, the modules are connected to each other by means of two sleeves placed one inside the other. Before separating, they are secured by a collar placed on the upper sleeve. The PCB is made of double-sided 1.54 mm thick laminate with an SMB1251B1-3GT30G-50 socket.

From the active surface, the galvanic plate is connected by means of four leads with a specific electrode made of electro-conducting silicone coated on the one hand in the galvanization process with a copper layer. Pins and the copper layer allow contact between the PCB and the electrically conductive silicone.

The specific electrode made of flexible electrically conductive silicone improves contact with the surface of the tested object. This feature is particularly useful in examining objects with increased porosity.

The third module is the tripod electrode mounting system. It is made of ASB in 3D printing technology. The holder has two parallel channels that allow quick mounting on tripod profiles. The element responsible for the elasticity of the mount is the rubber ring, which task is to adjust the position of the electrode to the tested object surface, which aims to eliminate the unevenness and pressing the electrode to the wall. The post-retrofit version is equipped with an additional 10 mm thick shock absorber and a flexible connection between the conductive rubber and the PCB. As a result, the electrodes adhere much better to uneven surfaces of the tested object. Newly designed electrode systems have great potential in practical applications.

### 2.2. Wall Moisture Tests with the Use of the Gauss-Newton (GNM) Method

**Figure 8:**
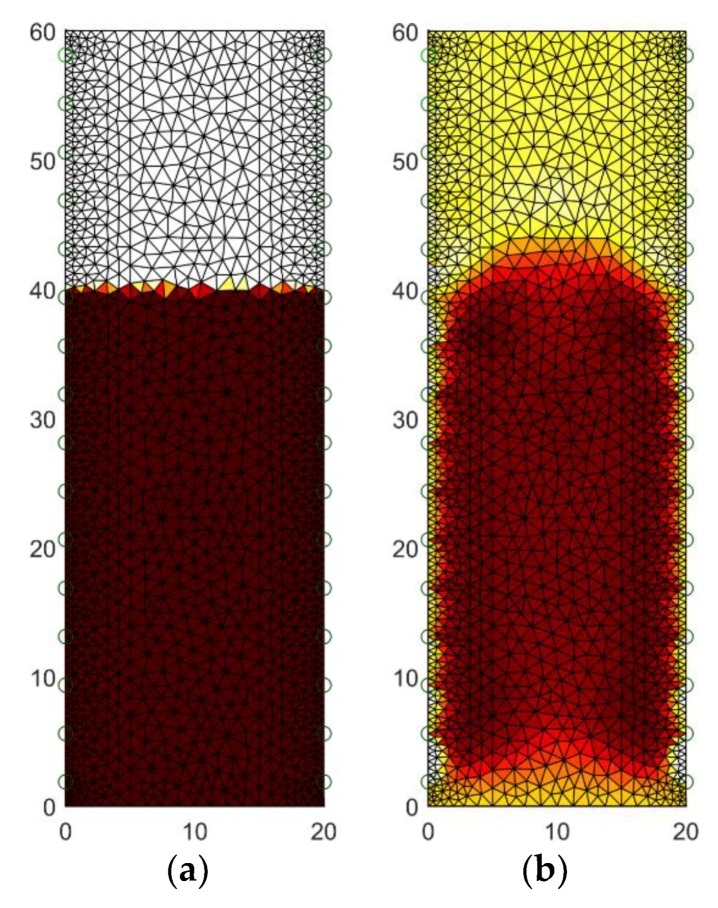
The geometrical model of the tested wet wall with 32 electrodes: (**a**) the pattern image, (**b**) the image reconstructed by Gauss-Newton method.

Figure 8 presents the results of tomographic imaging using the Gauss-Newton (GNM) method. The presented reconstruction (b) deviates somewhat from the pattern image (a). The differences, however, concern only the details of the contour of the moistened area. You can therefore use this method to roughly estimate the moisture level and area.

**Figure 9:**
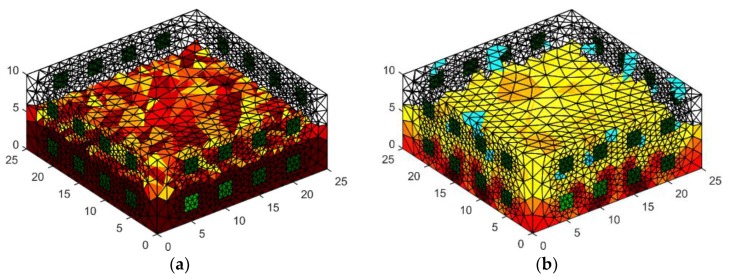
The geometrical model 3D with 4 × 8 electrodes—the image reconstruction: (**a**) pattern model, (**b**) Gauss-Newton method with Laplace regularization.

Figure 9 presents the spatial reconstruction of a wall fragment using the GNM method by means of 32 measurement electrodes located around the object. Figure 9a is a reference image. Figure 9b is the result generated by the use of GNM. Comparing both images, you can see differences in the intensity of the color. Brighter colors of the reconstructed image indicate less intense moisture inside the wall compared to the reference image.

**Figure 10:**
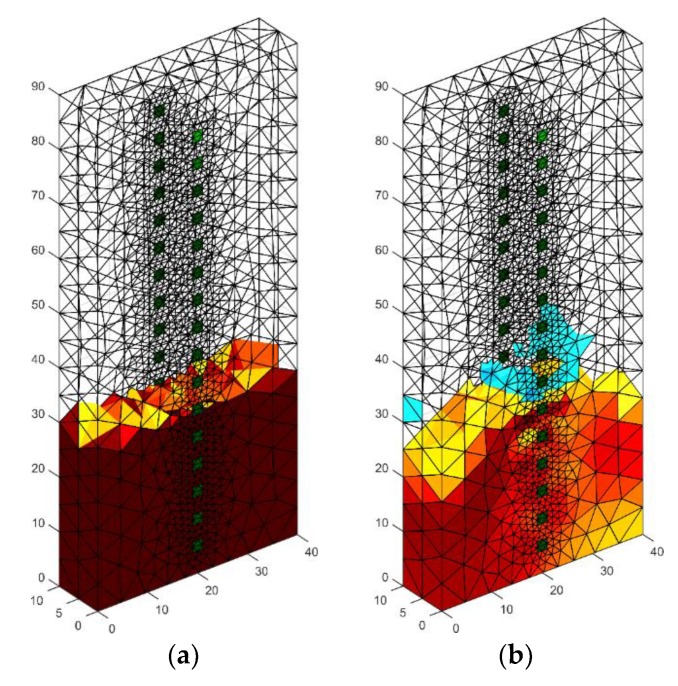
The geometrical model 3D with 2 × 16 electrodes—the image reconstruction: (**a**) pattern model, (**b**) reconstruction created with the use of Gauss-Newton method with Laplace regularization.

In Figure 10, we can see an example of a reconstruction of a damp concrete block using 32 electrodes located on both sides of the tested object. In order to solve the problem of the three-dimensional finite element, mesh was prepared. It can be noticed that surfaces of finite elements that are localized near electrodes are small. Hence, the solution of the forward problem is precise. The results obtained are similar to those obtained by placing 32 electrodes around the lightweight concrete block with dimensions 10 × 40 × 90 cm. The reconstructed image deviates from the pattern with the too low intensity of colors.

**Figure 11:**
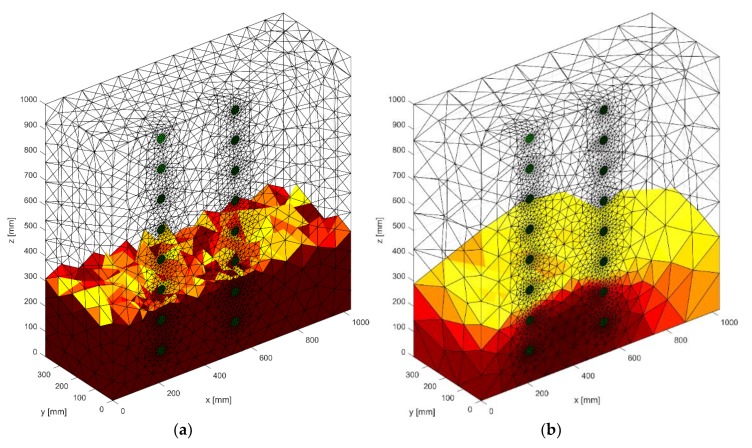
The geometrical model 3D with 2 × 8 electrodes—the image reconstruction: (**a**) pattern model, (**b**) reconstruction created with the use of Gauss-Newton method with Laplace regularization.

In Figure 11, two special models of the brick cube “wet” and “moist” with 2 × 8 electrodes are presented. The image was reconstructed by Gauss-Newton method with Laplace regularization or Tikhonov regularization.

### 2.3. Masonry Humidity Testing by the Least Angle Regression (LARS) Method

In order to obtain more accurate and stable reconstruction results in solving the inverse problem in electrical tomography [19,20,21], a new solution based on the method of the least angle regression was tested [27]. There are many methods to solve the optimization problem [26]. The statistical methods can be used to reconstruct an image in electrical impedance tomography [28,29].

The main objective of the tomography is to perform image reconstruction. During the measurements, we can see that the measured values from some electrodes are strongly correlated (due to the way of measurement). In this case, we have a multicollinearity problem. When the independent variables (predictors) are correlated (collinear), the matrix tends to a single matrix. By means of the least squares method, we obtain large absolute values of some estimators with unknown parameters. Forecasts based on this model are unstable. The most common approach is to reduce the set of input variables (removing the same predictors that apply to multicollinearity). Then, we have a problem with the selection of predictor variables that will be included in the regression model. For example, when comparing the AIC (Akaike Information Criterion) value for linear models with different sets of predictors, we can choose the best model.

Another possible way to reduce the problem of multicollinearity between predictors depends on the application of the least angle regression algorithm. This algorithm takes into account only causal variables in the linear model (from the set of predictors, you should select the input variables that have a direct impact on the response variable). In this case, the linear model is built by means of step forward regression, where the best variable is added to the model in every step.

Let the linear system be described by the state equation

(1) Y=Xβ+ε 

where $Y\in R^{n},\;X\in R^{n\times \left(k+1\right)}$ denote the observation matrices of response and input variables, respectively, and $\beta \in R^{k+1}$ denotes the vector of unknown parameters. When the linear model (1) contains the intercept, then the first column of matrix *X* is a column of ones. The object $\varepsilon \in R^{n}$ in the linear system (1) presents a sequence of disturbances, which is usually defined as a vector of independent identically distributed random variables with normal distribution $\;N\left(\tilde{O},\sigma^{2}I\right)$, in which $\tilde{O}\in R^{n}$ is a zeros vector, but $I\in R^{n\;\times \;n}$ is an identity matrix. The classical Least Square Method depends on identification of unknown parameters $\beta =\left(\beta_{0},\beta_{1},\ldots ,\beta_{k}\right)$ in (1) by solution the task

(2) $$\underset{\beta \in R^{k+1}}{\min}{\left\|Y-X\beta \right\|}^{2}\;$$

If $\det\left(X^{T}X\right)\neq 0$, then the best unbiased linear estimator of unknown parameters β is

(3) $$\hat{\beta }={\left(X^{T}X\right)}^{-1}XY\;$$

The problem is often when $X^{T}X$ is singular. 

The following is a short version of the least angle regression algorithm as the workflow. An extended version of LAR has been presented in [30].

1. The predictors should be standardized. The intercept $\beta_{0}$ in expression (1) is equal a mean of the response variable and we put $\beta_{1}=\beta_{2}=\ldots =\beta_{k}=0$. Active set *A* (set of predictors) is empty.
2. Calculate the residuals $r=Y-\beta_{0}-X_{\left(A\right)}\beta_{\left(A\right)}$ for the linear model with all predictors from active set A. Determine the predictor *X_j_* (which is not in active set) most correlated with residuals *r* and attach to the active set *A*.
3. Move coefficient $\beta_{j}$ from 0 towards its least-squares coefficient $\langle X_{j},r\rangle$ until some other competitor $X_{k}$ has a much correlation with the current residuals as does $X_{k}$.
4. Move $\beta_{j}$ and $\beta_{s}$ in the direction defined by their joint least square coefficient of the current residual on $\langle X_{j},X_{s}\rangle$ until some other competitor $X_{l}$ has a much correlation with the current residual.

Go to step 2 and continue in this way until all *k* predictors have been entered.

### 2.4. Masonry Humidity Testing by the ElasticNet Method

Another way to determine the linear regression when the input variables are collinear depends on the solution of the task

(4) $$\underset{\left(\beta_{0},\beta^{\prime }\right)\in R^{k+1}}{\min}\frac{1}{2n}\sum_{i=1}^{n}{\left(y_{i}-\beta_{0}-x_{i}\beta^{\prime }\right)}^{2}+\lambda P_{\alpha }\left(\beta^{\prime }\right),\;$$

where $x_{i}=\left(x_{i1},\ldots ,x_{ik}\right)$, $\beta \prime =\left(\beta_{1},\ldots ,\beta_{k}\right)$ for 1≤i≤n and $P_{\alpha }$ is an elastic net penalty given by

(5) $$P_{\alpha }\left(\beta^{\prime }\right)=\left(1-\alpha \right)\frac{1}{2}{\left\|\beta^{\prime }\right\|}_{{}_{L_{2}}}+\alpha {\left\|\beta^{\prime }\right\|}_{{}_{L_{1}}}=\sum_{j=1}^{k}\left(\frac{1-\alpha }{2}\beta_{j}^{2}+\alpha \left|\beta_{j}\right|\right)\;$$

We see that the penalty is a linear combination of norms $L_{1}$ and $L_{2}$ of unknown parameters β′. The introduction the penalty function dependent from parameters to the objective function allows to shrink the estimators of unknown parameters.

The parameter λ in the task (4) denotes the coefficient of penalty, but the parameter 0≤α≤1 creates the compromise between LASSO (Least Absolute Shrinkage and Selection Operator) and ridge regression. The ridge regression (∝=0) is called Tikhonov regularization [31] and is one of the most commonly used for regularization of linear models. LASSO (∝=1) was introduced by Roberta Tibshirani [32,33]. This method performs the variable selection and regularization in linear statistical models [34,35]. For the ridge regression, the penalty is calculated in the norm $L_{1}$ but for LASSO in $L_{2}$. Difference between ridge regression and LASSO is symbolic, only the norms are changed. The ridge regression shrinks coefficients for correlated predictors towards each other. When the correlated predictors depend on any latent factor, then ridge regression allows to uniformly distribute the strength of latent factor on these predictors. Whereas LASSO is indifferent to correlated predictors. This method allows to determine the preferred predictor and to ignore the rest. By applying the LASSO method, we obtain a model, where the many coefficients to be close to zero, and as a result, we receive a sparse model. The elastic net is a connection of ridge regression and LASSO [36,37]. Choosing the appropriate α, we may create the compromise between ridge regression and LASSO.

By solution the task (4) for fixed λ and α we estimate the unknown parameters of the linear system (1), where predictors are correlated. Then the prediction based on model (1) is given by the formula $\hat{Y}=X\hat{\beta }$, where the vector of estimators of unknown parameters $\hat{\beta }=\left({\hat{\beta }}_{0},{\hat{\beta }}_{1},\ldots ,{\hat{\beta }}_{k}\right)$ is estimated by solution the task (4).

### 2.5. Masonry Humidity Testing by the Gauss-Newton Method

In the electrical impedance tomography in the reconstruction of the image, the so-called Generalized Tikhonov regularization is very often used. In the literature on the subject, this method is also known as the Gauss-Newton algorithm in a generalized form.

The Gauss-Newton method is based on the application of the least squares method in which the matrix $Z^{\left(l\right)}$ fulfills the role of matrix X (first partial derivatives relative to fixed approximations $\beta^{\left(l\right)}$ and observed values of independent variables), and the role of the vector ***y*** (observation of the dependent variable) vector $e^{\left(l\right)}$. It is a vector of differences between the empirical values of the dependent variable and the lth of its approximations $f\left(x_{t},\beta^{\left(l\right)}\right)$.

The Gauss-Newton algorithm is used to estimate the structural parameters of non-linear models. The general form of the non-linear function is presented below:

(6) $$\;y_{t}=f\left(x_{t},\beta \right)+\varepsilon_{t}\;$$

where:

$y_{t}$—observations of the explanatory variable,
$x_{t}=\left[x_{t}\right]$—P vector of observations for explanatory variables,
$\beta_{t}=\left[\beta_{j}\right]$—K vector of structural parameters,
$\varepsilon_{t}$—implementations of random elements (we assume that random components are uncorrelated, have an average of zero and equal, positive and finite variance).

In the Gauss-Newton method, the reconstruction of the internal image of the investigated object is related to the determination of the global minimum of the fitness function. In order to carry out quantitative considerations, we assume that the tested object is polarized with an alternating low-frequency current. Then, the electrical material properties can be described by a function with real values. In this case, in the generalized Laplace equation, we neglect the word proportional to the frequency, and this function can be equated with the electrical conductivity (real isotropic admittivity case).

### 2.6. Masonry Humidity Testing by the Neural Imaging

In order to solve the problem of non-invasive imaging of the interior of moist walls, the method of electrical tomography in connection with artificial neural networks was also used. So far, tomographic and neural networks methods have not been widely disseminated in the assessment of the wall. The reason is the low resolution of the reconstructed image and the low accuracy of mappings [7].

To increase the resolution of tomographic reconstructions depicting the degree of internal humidity of walls, a new method was developed based on a set of many separately trained neural networks. The number of neural networks corresponds to the 3D resolution of the lattice dividing the inside of the wall into individual pixels. In the presented experiment a lightweight concrete block with dimensions 10 × 40 × 90 cm was used, which was divided into 8099 points.

Using a device called a multiplexer, in short intervals, the tomographic system generates 192 values of voltage drops readings between different electrode pairs. These are the input data for the neural network system. The neural networks are designed in such a way that on the basis of an input vector containing 192 elements, each of the 8099 neural networks generates the value of a single pixel of the output image.

**Figure 12:**
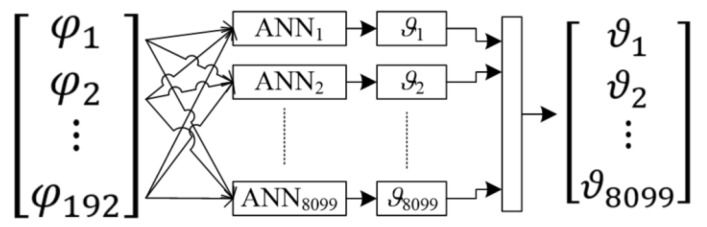
The scheme of converting electrical signals into the image pixels.

Figure 12 shows the mathematical form of the neural model used during simulation experiments. At the model input, there are 192 electric signals generated by 16 electrodes.

**Figure 13:**
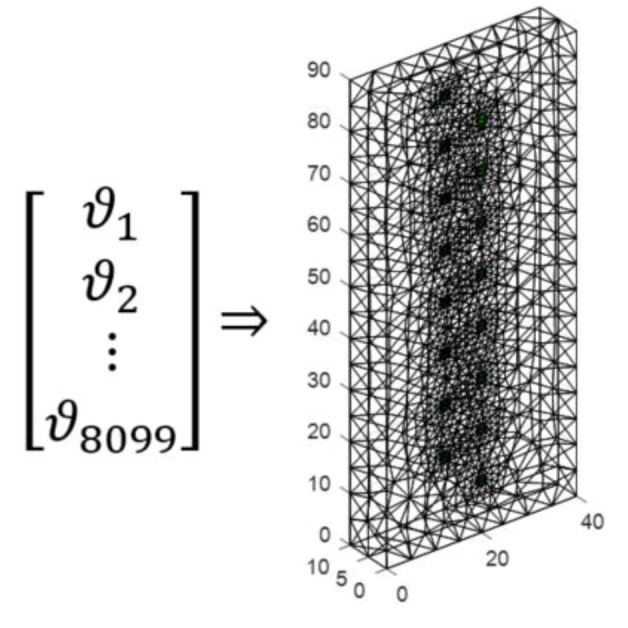
The way of converting a real number vector into the lightweight concrete block’s spatial image.

The same input vector is the basis for training 8099 separate artificial neural networks (ANN). In this way, from a vector of 192 variables representing electrical values, a set of neural networks creates a complete lattice of the lightweight concrete block image. The output image is created by assigning colors to the output values of each pixel. The transformation method is shown in Figure 13.

**Figure 14:**
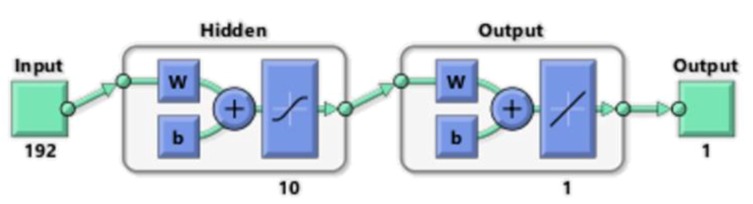
Structure of the selected multi-layer perceptron.

Each of the 8099 neural networks had a multi-layered perceptron structure with 10 neurons in the hidden layer. The scheme of a single perceptron is shown in Figure 14.

In order to collect data necessary to train the neural network, physical and mathematical models were developed. The finite element method was used for this. Based on a mathematical model, a data set was generated. After that, it was used to train the neural network system.

To train the mentioned above neural network, a collection of 6140 historical cases was used (see Table 1). The main set of data has been divided into three separate subsets: a training set, validation set, and testing set, in the proportions of 70%, 15%, and 15%. This method of data preparation has been used for all 8099 neural networks.

The highest Mean Squared Error (MSE) concerned the testing set and was 0.000020364. In the case of validation set, a slightly smaller error was noted. Mean Squared Error is the average squared difference between outputs and targets. Lower values mean better performance. Zero means no error (excellent performance). The training set was trained with the lowest training error, which is the most common and correct situation. A low MSE error in the training set results from better network adaptation to training cases. Another indicator of the quality of network learning was R (Regression). An R value of 1 means a close relationship between pattern and output, and 0 means a random relationship. In all three cases of data sets (learning, validation, and testing), R was close to 1. This also applies to the test and validation set, which is particularly valuable. Values close to 1 indicate a good match of the results obtained by the network (output vectors) to the patterns included in the individual sets (training, validation, and testing).

Good indicators (MSE and R) for the training set show the lack of overtraining effect and the ability of the network to knowledge generalization (i.e., correct conversion of input data to output information not only for learning cases).

## 3. Results

This chapter presents the results of wall humidity tests by EIT tomography in combination with the following machine learning algorithms: Least Angle Regression (LARS), ElasticNet, and Artificial Neural Networks. The root mean square error of prediction (RMSE) indicator was used to quantify the quality of the reconstructions obtained using simulation models.

Let vector $x=\left(x_{1},\ldots ,x_{n}\right)$ presents the pattern, which should be reconstructed. After reconstruction we obtain the vector $\hat{x}=\left({\hat{x}}_{1},\ldots ,{\hat{x}}_{n}\right)$, which contains the expected values of reconstruction of explored object. The root mean square error of prediction was determined by the formula (7).

(7) $$RMSE=\sqrt{\frac{1}{n}\sum_{i=1}^{n}{\left(x_{i}-{\hat{x}}_{i}\right)}^{2}}\;$$

In the further part of this paper two variants of images were compared: 2D and 3D. The 2D image lattice consisted of 2908 pixels, while the 3D grid consisted of 8099 pixels. Thus, in the first case (2D), *n* = 2908, while for the 3D variant, *n* = 8099.

All results enabling the comparison of LARS, ElasticNET, and ANN methods were obtained thanks to the use of computer simulation methods. The simulated data has been used with added noise to SNR = 14 dB. This noise parameter was calculated for a given measurement system configuration and includes measurement patterns and electrode positions. SNR is defined in terms of power as a signal to noise ratio.

### 3.1. Results of Wall Moisture Tests Obtained Using the Least Angle Regression (LARS) Method

**Figure 15:**
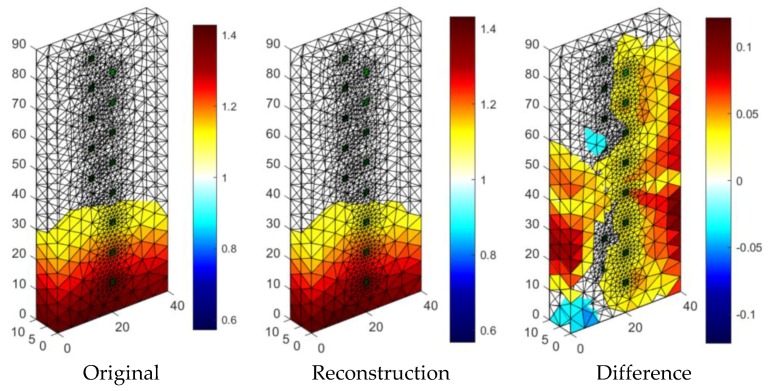
The result of Least Angle Regression (LARS) moisture testing of the lightweight concrete block for the case of 2 × 8 electrodes.

Figure 15 presents one of the results of tomographic imaging using the LARS method. The input data was obtained thanks to the use of an EIT tomograph equipped with 16 electrodes (2 × 8). Intense colors indicate areas with higher humidity. It can be seen, the obtained reconstructive image (in the middle) is very close to the reference image (left). The difference image (right) indicates small deviations of the grid points in the reconstructed image from the reference image. The colors in the images reflect the conductance of the individual pixels that each image consists of. The lack of color in the original and reconstruction images testify to the lack of moisture. RMSE for a 3D sample with the use of LARS is 0.0298.

**Figure 16:**
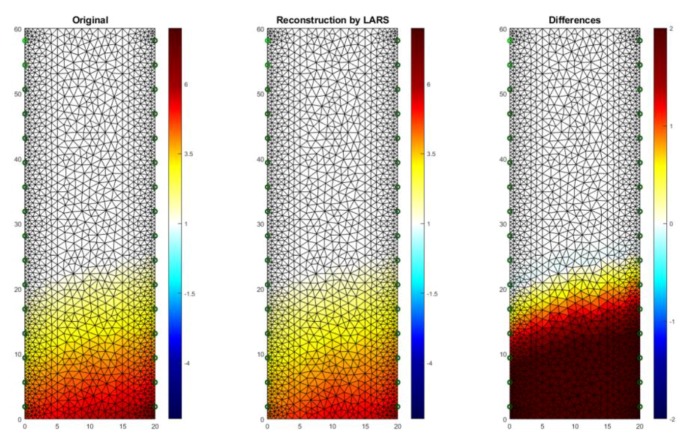
The result of Least Angle Regression (LARS) moisture testing of the lightweight concrete block for the case of 2 × 16 electrodes.

Figure 16 shows a case analogous to the previous one, which was presented in Figure 15, but this time a cellular concrete sample with slightly different dimensions (10 × 20 × 60 cm) was used. The reconstruction was carried out in 2D. RMSE for a 2D sample with the use of LARS is 0.122599.

### 3.2. Results of Wall Moisture Tests Obtained Using the ElasticNet Method

**Figure 17:**
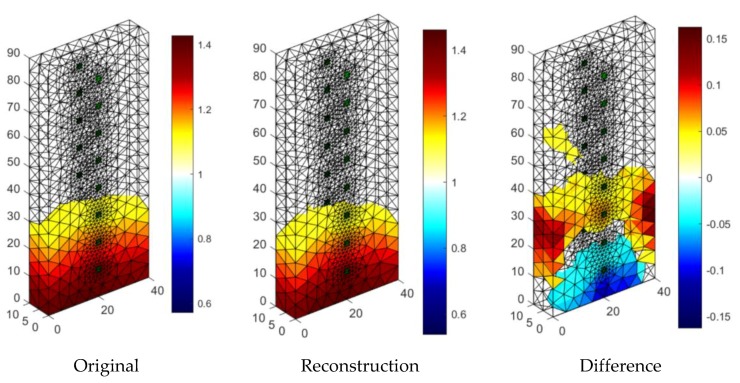
The result of ElasticNet moisture testing of the lightweight concrete block for the case of 2 × 8 electrodes.

Figure 17 presents an example of a tomographic 3D imaging result using the ElasticNet method. The input data was obtained thanks to the use of an EIT tomograph equipped with 16 electrodes (2 × 8). Intense colors indicate higher humidity spots. It can be seen, the obtained reconstructive image (middle image) is comparable to the reference image (left). The image of residuals (right) indicates the occurrence of deviations of the grid points of the image reconstructed from the reference image. The lack of color and shades of blue in the original and reconstruction images mean the lack of moisture. RMSE for a 3D sample with the use of ElasticNet is 0.0365.

**Figure 18:**
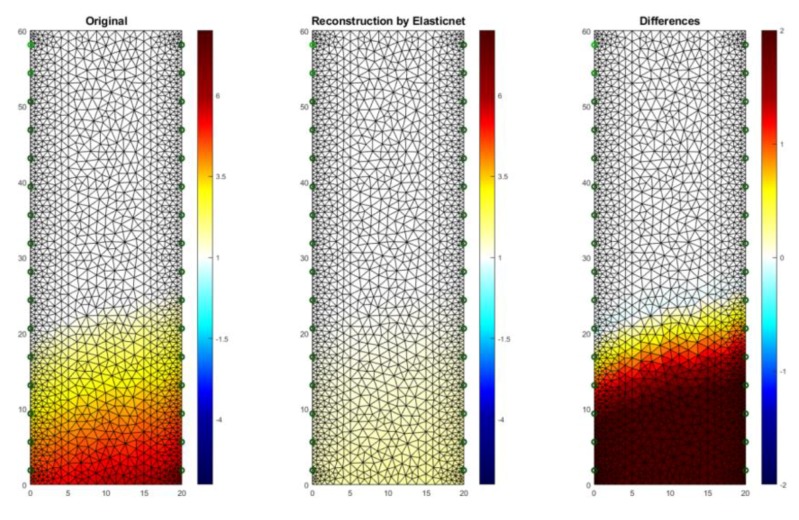
The result of ElasticNet moisture testing of the lightweight concrete block for the case of 2 × 16 electrodes.

Figure 18 presents an example of a tomographic imaging result using the ElasticNet method. The input data was obtained thanks to the use of an EIT tomograph equipped with 32 electrodes (2 × 16). Intense colors indicate higher humidity spots. It can be seen that the obtained reconstructive image (middle image) reproduces the reference image (left) poorly. The image of differences (right) indicates the occurrence of significant deviations of the grid points of the image reconstructed from the reference image. RMSE for a 2D sample with the use of ElasticNet is 0.282520. Compared with LARS, ElasticNet showed the lower quality (higher RMSE) of reconstruction in this case.

### 3.3. Results of Wall Moisture Tests Obtained Using the Neural Imaging

**Figure 19:**
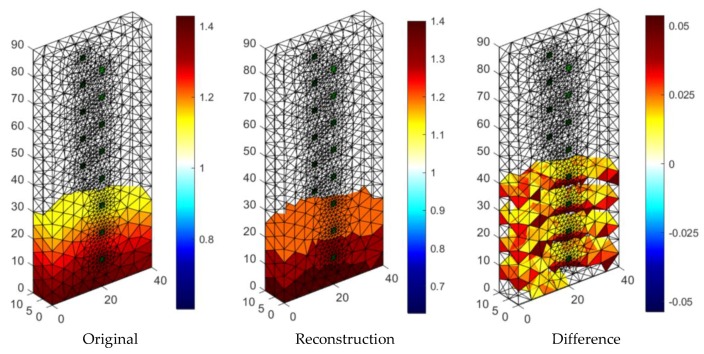
The result of Artificial Neural Network system (ANN) moisture testing of the lightweight concrete block for the case of 2 × 8 electrodes.

Figure 19 presents the results of neural imaging in conjunction with the EIT. Conductive (positive) areas are shown in shades of red. Non-conductive (negative) areas are shown in blue or they are transparent. Comparing the pattern of the damp block (original) with the output image, we conclude that the accuracy of the imaging is very high. The right image shows the absolute (numerical) differences in the values of individual pixels are minimal. They do not exceed ±0.05. It can be seen that the results obtained by the neural imaging method are comparable to the results obtained by both previous methods. RMSE for a 3D sample with the use of ANN is 0.010819 so the quality is better than LARS and ElasticNET.

**Figure 20:**
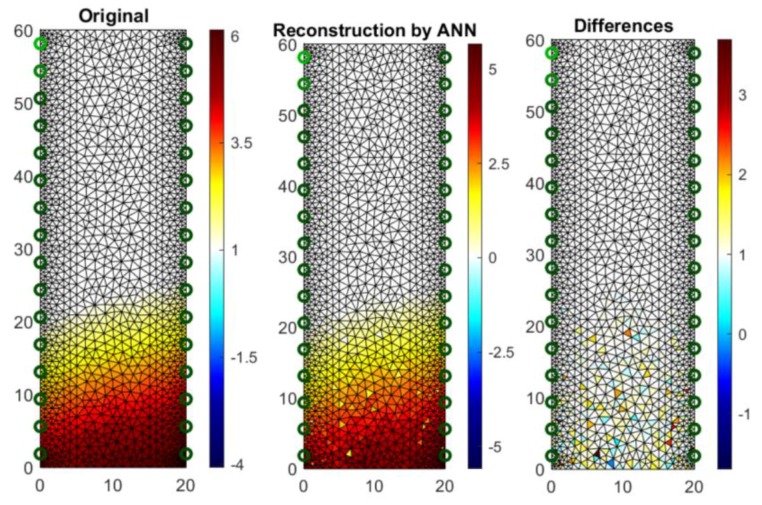
The result of Artificial Neural Network system (ANN) moisture testing of the lightweight concrete block for the case of 2 × 16 electrodes.

Figure 20 shows an analog measurement of a 2D sample with two rows of electrodes on both sides of the block (2 × 16). Noteworthy is the small amount of colored pixels in the differential image. This indicates high quality imaging, which is confirmed by the low RMSE index, which in this case equals 0.106301.

### 3.4. Moisture Test of Real Object

**Figure 21:**
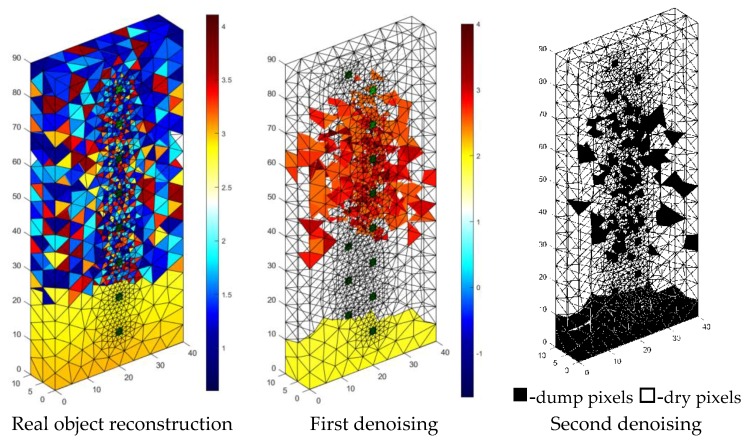
The result of the real object Artificial Neural Networks (ANN) moisture testing of the lightweight concrete block for the case of 2 × 8 electrodes.

Figure 21 shows the results of the reconstruction of a block of cellular concrete immersed in water, using a system of artificial neural networks. Reconstructions based on data obtained from real objects, in contrast to simulation experiments based on numerical models are the most difficult type of test for tomographic systems. The first image on the left is the result of the direct processing of the tomographic data with the use of ANN. The ambiguous visual effect is caused by noise in the value of the input vector, which in the case of real objects is basically unavoidable.

To show the results in a way that visually identifies the moisture area, the input vector was subjected to a denoising procedure using denoising stacked autoencoders [38]. The results of denoising were presented in the middle image entitled “First denoising.” Finally, the output image was subjected to one more processing using a filtering script whose objective was to cut off the output values, which obviously exceeded the acceptable range. The filtering effects are shown in the image entitled “Second denoising.” Finally, it was possible to reduce the number of incorrectly reconstructed pixels in the central part of the sample, but it was not possible to obtain a “clean image”.

Denoising of tomographic data is an important issue because it affects the results of reconstruction. Due to the complexity of this subject, it may be the subject of separate studies aimed at improving the quality of tomographic images.

## 4. Discussion

The imaging results presented in the previous chapter show great application possibilities of the machine learning algorithms combined with EIT. The analysis involved three methods and algorithms converting input vectors (values of voltage drops) into reconstructed images reflecting the conductance: Least Angle Regression (LARS), ElasticNet, and Artificial Neural Networks (ANN). Of the above methods, the best results were obtained using ANN. However, the LARS method in terms of fidelity representation is very similar to ANN.

Table 2 presents a summary of the RMSE values for cases of 2D and 3D imaging in relation to 3 methods tested: ANN, LARS and ElasticNET. The lowest values of the indicators, demonstrating the highest imaging quality, were obtained for ANN.

Both LARS and ANN can be successfully used in the EIT tomography dedicated to the reconstruction of moisture in masonries and building walls. In comparison to other, previously used algorithms, these methods allow obtaining precise images with sufficient resolution to perform an effective and error-free analysis of the moisture content of the walls. It is worth noting that taking into account the possibilities of spatial image creating, the LARS and ANN methods are more reliable than invasive methods requiring the sampling of masonry.

It is also important that the presented algorithms, used in connection with the EIT system and specially designed electrodes, have large application possibilities. Their basic advantages are functionality, reliability, measurement accuracy and reasonable price. The method is universal due to the possibility of applying to masonry and walls of the various structure, thickness and moisture level. An important role is also played by the speed of the computed tomography scanner. Output images are obtained in real time.

The electrical impedance tomography proposed in this article enables the creation of a new non-invasive technique for measuring the humidity of building walls. The EIT has been used to determine the conductivity distribution in specially constructed wall models made of light concrete blocks or bricks. The finite element method implemented in the EIDORS environment has been used to solve the problem. The numerous different lattices were used in the presented numerical models. The analyzed measurement systems contained various electrode distributions. Thanks to this, it is sure that the obtained results are not accidental but repeatable while maintaining similar conditions of the measurement environment.

The research has provided new and promising results. Future work will be continued thanks to the use of regularization techniques in the optimization process and the hybrid measurement system. These types of hybrid measuring system should be even more reliable in practical applications. It would also be interesting to extend the experimental measurements over time by monitoring the walls at regular intervals. Thanks to this, it would be possible to estimate the speed of spreading the moisture inside walls, as well as its sources and propagation directions.

## 5. Conclusions

The main goal of the work was to analyze the solution based on electrical tomography to study the moisture of walls. Non-destructive methods and algorithms have been analyzed and compared, which allow estimation of humidity also inside the wall. A new concept of a non-destructive system based on electrical tomography has been presented. For research purposes, specially designed electrodes were used, which were placed on the tested lightweight concrete and brick blocks. Three machine learning algorithms were tested: Least Angle Regression (LARS), ElasticNet, and Artificial Neural Networks.

It was found that all four methods are suitable for practical applications in EIT tomography dedicated to the detection of moisture in building walls, however, the best results were obtained using the LARS method and the specially designed multi-ANNs system. A characteristic feature of the analyzed solution is the division of the modeled object using a specially developed mesh for a set of elements. The color of each individual mesh element corresponds to the conductance value (in the EIT tomograph). Thanks to this approach the number of information determining the reconstructive picture was large enough to guarantee a sufficient resolution of tomography imaging.

The presented research results contain relevant information that may contribute to the acceleration of the development of computational intelligence and machine learning methods in EIT. The research contributes to the improvement of the tomographic imaging efficiency of known methods in the aspect of algorithms for processing input information (electrical quantities) into images. In addition, enriching an input vector with values other than electrical is an easy way to develop new, intelligent tomographic hybrid systems.

## Figures and Tables

**Table 1:** Training results for one of 8099 neural networks.

	Samples	MSE	R
Training set	4298	5.31979 × 10^−6^	9.99983 × 10^−1^
Validation set	921	1.68249 × 10^−5^	9.99947 × 10^−1^
Testing set	921	2.03645 × 10^−5^	9.99934 × 10^−1^

**Table 2:** Comparison of the quality of different imaging methods.

Method	RMSE	
	2D Samples	3D Samples
ANN	0.106301	0.010819
LARS	0.122599	0.029800
ElasticNET	0.282520	0.036500
